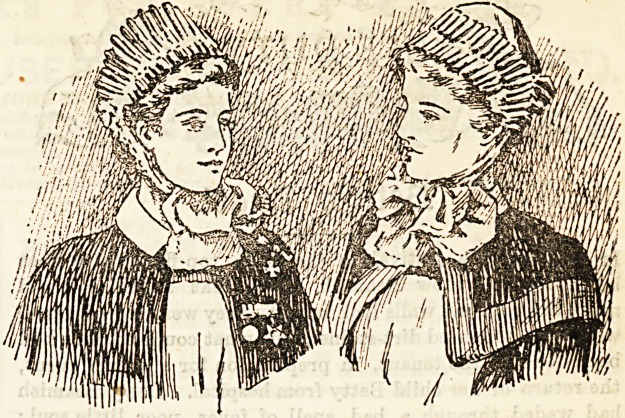# The Hospital Nursing Supplement

**Published:** 1892-03-05

**Authors:** 


### "The Hospital" Nursing Mirror.

Being the Extra Nursing Supplement of "The Hospital" Newspaper.

Contributions for this Supplement should be addressed to the Editor, The Hospital, 140, Strand, London, W.C., and should have the word "Nursing" plainly written in left-hand top corner of the envelope.

### En Passant.

WHY HOSPITALS ARE POOR.—Speakers on the chronic indigence of our hospitals, treatises, and lectures on the subject have not noticed the chief reason of the poverty complained of—the porter. He is usually a surly man, who receives all visitors with suspicion, who treats them in an off-hand manner, who thwarts and turns them away when they come with help in their hands. Again and again we have heard stories of a visitor arriving at an institution at an inconvenient moment, and being treated curtly and left waiting in cold corridors. Of course there must be rules in a big hospital, and there must be fixed hours; but the outside public, especially the rich and idle classes, do not always understand this, and the fact ought to be explained to them with courtesy, and a little time and attention given to them, even if they pour out sentimental twaddle. Those who give their money to hospitals have at least the right to be treated politely by the officials. We would request all Matrons to take steps at times to test the manners of the porters to strangers; we would ask them by their own example to teach a lesson of courtesy and consideration to all. Then visitors arriving in a fit of generous sentiment would not so often drive away with injured dignity and unemptied purses.

POOR-LAW NURSES.—The first expression of opinion we have heard on the new district nurses under the Poor Law was from Mr. Bryant, speaking at Westminster Hospital on February 24th. He said he believed the scheme would be both beneficial and economical, and that he hailed with feelings of pleasure any means of extending district nursing. On one point Mr. Bryant was at fault, so far as most unions are concerned. He said the general effort was to keep people out of the workhouse. Those guardians who do not believe in out-relief try to force people into the house. On January 1st, 1875, there were 1,799 cases of out-relief in St. George's-in-the-East, and the Board took vigorous measures, and at the end of the year there were only 548 cases of out-relief; but the paupers in the house were more numerous. In 1888 the Chairman of the St. Neots Board wrote: "We have granted no permanent out-relief for years." In parishes ruled in this way even the medical officer has little to do save in the infirmary. Mr. Mackay says, in his book on "The English Poor": "It seems best for guardians to offer the infirmary in sick cases a more adequate, but less eligible, form of relief." To guardians holding these principles the thought of district nurses for out-door paupers must be very objectionable, and we are afraid Mr. Bryant's opinion in favour of the scheme will not find a wide echo in union board-rooms.

CLEAN HANDS.—A lady lately asked a nurse from a large hospital to dine with her. When the nurse, who was in uniform, had put on her cap, the lady noticed that her fingers were grubby, her nails dirty, and rang for some warm water; but the nurse merely dabbled her finger-tips in the water, and went to table with hands the sight of which quite spoilt her neighbour's dinner. Next day one of the surgeons of the hospital called on the lady, and to his great disgust had the above little incident confided to him, the narrator ending up with: "But I suppose nurses all have dirty hands; I hear they have such menial duties to perform. In my young days ladies stayed at home and tried to fulfil the Bible injunction of clean hands and a pure heart." It was no use for the surgeon to try and make his friend believe that nurses were not habitually faulty in this respect; one of their number had brought disgrace on the whole profession. At an examination at a London hospital some time ago, as each nurse entered the room, she was requested to wash her hands; some looked indignant, and daintily dipped their fingers in the water; but one nurse, in answer to the request, took off her cuffs, turned up her sleeves, plunged her hands into the water, and used the soap and nail-brush freely. She was the oulv nurse that passed that examination! Both the above incidents are true, though we know that many of our readers will have some difficulty in believing that a nurse by profession, a lady by birth, could so disgrace her fellow-workers as to go about with dirty hands and nails.

SHORT ITEMS.—On February 23rd the first concert in the Male Convalescent Home, belonging to the county of Surrey, was given. The performers were the Matron, Miss Napper, and several of the patients. A most enjoyable evening was spent.—By mistake we stated last week that the new institution at Acton was started by Mr. instead of Mrs. Gordon Elliott; the institution has a good chance of success.—A delightful and practical course of lectures by a Hospital Sister is being given just now at Adelphi Terrace to Mrs. Selfe Leonard's nurses and Bible women.—Kinsale Guardians have got an unsatisfactory state of affairs at the workhouse, and proposed to introduce nuns to remedy it. The proposal has been lost, however, and the Guardians are now going to appoint trained nurses.

AMONGST THE LEPERS.—In last week's *Woman's Herald* was a letter from Miss Conybeare describing a visit she paid to the leper settlement on Robben Island. She says: "Two years ago the lepers on this island were in very wretched quarters, and few took any interest in their sad condition—since then, chiefly due to the exertions of a lady, attention was attracted to the whole question—new buildings were erected, properly trained nurses engaged, and now they are well cared for. It was Christmas week, and the lepers, with the assistance of the nurses, had decorated their rooms most gaily, festoons of coloured paper, Japanese lanterns, and flags canopied the different wards. On the walls were the large coloured pictures from all the various Christmas extra numbers. Wild asparagus and other greenery gave the whole scene a most festive aspect. I was agreeably surprised to find how cheerful the lepers were; many with their fingerless hands were nevertheless playing draughts or other games. Some were reading, others had slates and were practising writing, for the chaplain holds classes for those who like to learn. They were chiefly coloured people, though among them were a few white men. The children were the most terrible sight. Little boys of ten and twelve whose faces were swollen and disfigured till they reminded you of pictures of ogres, their enormous heads, and small stunted bodies, gave them the appearance of old dwarfs. The two young nurses were evidently great favourites among their patients. One had been trained at Manchester, the other at Leicester. Both were pretty, with fair hair, and in their white caps and uniforms they were pleasant objects amidst so much that was necessarily hideous and painful to the eye. They told me that they were not at all afraid of catching leprosy except by inoculation, and they were very careful not to get cuts on their hands."

### On the Nursing of Children.

### IV.—OPERATION CASES (*continued*).

The nursing of babies with hare lip involves a great deal of trouble, and although it is only for a short time, the probationer who once longed to be entrusted with a case, seems after two or three experiences to have her appetite completely appeased.

It is essential that everything should be done during the days preceding the operation to bring the baby into as sound and healthy a state as possible, and great pains must be taken to ensure its getting a sufficient amount of nourishment, and this takes a great deal of time, if a cleft palate complicates the situation. Surgeons' opinions as to the best age to do the operation vary considerably; but from the nurse's point of view, the younger the baby the better she likes it. Supposing its general condition good and no sore, nor any sort of affection of any part of its skin to exist, the infant of a week or two old seems not only to bear the operation well, but to recover from it and to be the possessor of a "pretty" lip with surprising rapidity. The fact that it needs less nourishment and cries but feebly is, of course, in its favour. Still, as many surgeons prefer waiting till the baby is six, or even twelve, months old, the greater difficulty of nursing the stronger, older, and hungrier child must be surmounted. Whether the operation has been a simple or rather complicated one, of this we may be sure; with the return of consciousness, a pitiful wail will commence, and this must be soothed. If the doctor has ordered a draught, matters are simplified, and the little one will be quieted for some hours; but if he prefers to omit this, nurse has to work out the problem unaided, for the child must not be allowed to cry, although he has a perfect right to do so, being doubtless very uncomfortable.

It is probable that the poor little strapped-up lip does not annoy him at all at this stage, but he is upset by the chloroform and he feels generally miserable.

To feed a child directly it cries, is mistaken treatment at any time, as indigestion may be the cause of its fretfulness, and food should therefore be given carefully at regular times only, and, of course, never immediately after an operation. Warmth is the first object to be secured for the infant, who is probably chilly by this time, in spite of his blankets. If a fire be obtainable he should be nursed in front of it, and the little feet and legs exposed to its comforting influence, and warm cotton wool or a piece of new flannel can be satisfactorily applied to the uneasy little stomach.

A trained "children's nurse" has always a number of expedients to which has recourse when the first attempt fails. As regards the baby's hands a word may be said—muffling with fingerless gloves or pads of cotton wool, is not sufficient to prevent their doing mischief to the strapping when the lip begins to fidget them; it is better from the first to put it out of their power to do harm. If the body be satisfactorily clothed in a good knitted or flannel vest, or cotton wool jacket, the night gown should be without armholes or sleeves, and it should cross well over at the opening and be securely buttoned there, so that the tiny restless fingers cannot work out. Of course, a blanket, firmly rolled round the child pinions the arms, but it is the best in all cases to try whichever plan is least uncomfortable, so long as it fulfils the object desired.

As regards the feeding of hare-lip cases after the operation, it is always well to ask each surgeon what interval he wis hes to elapse before the first food is given, but if he leaves this to the nurse she can safely let a healthy child wait six hours at least, thus giving a good chance to the lip, for even the most experienced woman is liable to let a little moisture stray to the strapping from the teaspoon.

As regards tracheotomy, whether it be performed for a scalded glottis, for croup, diphtheria, or on account of the presence of "a foreign body," there are many general rules to be observed. Of the arrangement of the tent, of the steam-kettle and the thermometer, many nursing books and lectures give sound instruction, and the tube is the nurse's first thought, but there are other matters of no less importance which get less attention. First of all the fire; this should be kept good, never allowed to grow so low as to necessitate a "great making up." It should be constantly fed with small quantities of coal if need be, or at any rate so managed as to be always bright. If the steam kettle is filled with boiling water, there is no perceptible diminution in the cloud of steam, and if it is said "to go for four hours," it is generally desirable to fill it at the end of every three and a-half hours. The kettle which is kept on the fire, or better still, on a trivet, should be sufficiently large to contain the water required, but not too big, or it will spoil the fire needlessly, and whenever the steam kettle is filled the empty kettle should be immediately replenished. To neglect this, is a sign not only of a careless nurse, but of a selfish woman. A ward kettle standing empty in the fender is a disgrace, and should never escape notice. If boiling water be needed for a sick or wounded person, it is wanted suddenly, and there is no time to be wasted by a cold kettle being put on the fire when an emergency arises. And this applies to the nurse in charge of the tracheotomy case as well as to all other nurses and probationers. The thermometer which is placed conspicuously near the head in the tent, should be observed at regular intervals, say every hour, especially during the night. If the temperature has gone down, blankets must be added to the "tent cover" and foot-warmers put in the cot, and means taken speedily to remove the dangerous state of things. A locker or table, out of sight of the little patient, but conveniently near the nurse, should hold the nourishment which has to be administered, and the porringers, feathers, tubes, &c., should be on a different board or table. A stand for the kettle with its spirit lamp should be kept for that alone, and a neat nurse should manage to replenish these, without making any slop of water or spirit. Great care should be exercised in washing a child in a tent, for chills are specially dangerous to him, and the nurse must spare no pains in guarding against them.

There are so many operations of all kinds done every day that it is difficult to speak of one, without branching off into others, but as the chief points in the nursing of children are the same in all cases, we will leave the subject, and speak of splints and appliances in another article.

### Notes and Queries.

### Answers.

*Medicus.*—We cannot insert appeals unless from nurses connected with some institution, or unless the address is for publication.

*Pneumonia Jacket.*—An old vest cut open down each shoulder and down each side, and supplied with strings, makes a good jacket for keeping on pneumonia poultices. If the vest opens in front, sew it up there. Or cut flannel to similar shape.

*Nurse F. H.*—We have not used the information you sent, as we do not think your example a good one. It is hardly fair to the hospital, is it?

*Life Plant.*—Safely received and acknowledged to Bermuda, but in case the letter misses you there, this is to ask you to address all news in future to the Editor, at this office.

*Homes Wanted.*—Correspondents are requested to note that list of institutions and convalescent homes will be found in the "Hospital Annual," price 3s. 6d., from this office.

*Sister H.*—Many thanks; we shall be very pleased to help you when the time comes.

*Mot.*—For particulars concerning examinations for attendants, apply to the Secretary, Medico-Pyschological Society, Hanwell Asylum, W.

*Maud.*—(1) That depends, there are lectures. (2) Certificate at end of two years. (3) Salary fair.

*Katherine.*—You can take a certificate as monthly nurse in six weeks, but doctors do not care to employ a nurse who has not had at least three months; and, if possible, you should take the L.O.S. diploma.

*Veritas.*—We never prescribe; consult a medical man.

*Attendant.*—"Work in the Wards," by the Rev. H. Hawkins, price 1s. the dozen, published by the S.P.C.K.

### GREAT THOUGHTS.

In our daily life we seem to have no time for great thoughts, little ones fill our minds, and cares, troubles, aches, and pains crowd out the finer wishes of our natures. Ye we should be brighter and happier if we encouraged nobler views, and our characters and actions strengthened and improved by dwelling on higher things.

We all own that out of the fulness of the heart the mouth speaketh; if then we are filled with nought but vanity and vexation of spirit, will not our talk be seasoned with bitter or foolish words? Alas! yes; we cannot expect to get both sweet and bitter water from the same source. Some may ask, where are these wonderful thoughts to be found, which are to do such great things for us; do they crop up of themselves like weeds in a field? No, indeed, we must go to some store-house for them, where we shall find them lying all ready to our hand. These treasure houses are books, in which wise and learned men have written down their own thoughts and experiences for the benefit of those who are less clever than themselves. Everyone who can read—and which of us now-a-days cannot?—may choose for himself, but the best treasury we can go to is the Bible; there we shall see great and noble thoughts in endless profusion; they will serve us all our lives.

Take, for instance, the following: "God so loved the world, that He gave His only begotten son, that whosoever believeth on Him should not perish, but have everlasting life." Here is something for the weak and languishing to catch hold of. "Life." It is the very thing they are longing for, "Life" which seems ebbing away from them without the possibility of return. But here is the gift put within their reach; they may grasp it if they will. The loving Father has freely given this life, full, strong, endless, that will satisfy us through time and through eternity. And all we have to do is to cast away all doubting and mistrust, and to believe, each for himself, that He, the Life, was given for and to us. Can anything be more beautiful or comforting; to know that our Heavenly Father has such love for us, that He would not have the meanest of us to perish? How great, how stupendous the power which can make and govern worlds, and yet with condescension stoops to watch over the fowls of the air; and as we are so much better than they, as we have souls to be saved, so much greater is His care towards us.

When we have taken Christ for our own personal Saviour, peace and joy in believing naturally follow; the Life which flows from Him will fill us with strength; heart and mind, endued with new vigour, will grow more like Him day by day, and be fitted to serve Him better in time or in eternity.

This is, indeed, a great thought that God so loved the world and its inmates that He gave them the gift of eternal life.

### Notes from Australia.

(*By our Own Correspondent.*)

Melbourne, January 19th.

The enquiry into the conduct of the Matron of the Austin Hospital has ended in a quaint verdict of "Not guilty, but don't do it again;" or, in the words of the resolution: "That the Committee, after enquiring into the management of the Austin Hospital, is satisfied that the hospital is well managed, and that the charges made against the Matron have not been sustained. At the same time the Committee, while recording confidence in the management, expresses the hope that the Matron will exercise as much kindness and tact as possible in the discharge of her duties."

It will be enough to quote the following samples of the complaints brought forward. Twelve of the patients wrote: "The nursing arrangements are a long way from what they should be, as the various wards have been left at times without a nurse for days together, consequently the patients are neglected, especially the helpless ones, and patients have been heard to say that they prayed to God that they would be taken away before they became thoroughly helpless, and be subjected to the ill-usage and roughness that they have seen others subjected to. Also, we would wish to bring under your notice that our lives here might be made far more comfortable and happy if the Matron showed towards us a more kindly, Christian, and sympathetic feeling than that arbitrary, abrupt, and austere manner that she has hitherto done."

And here is the evidence of a lady cleric: "Miss Muir, the assistant chaplain of the Presbyterian Church, said that she was a weekly visitor to the place. There did not appear to be any sympathy between the Matron and the patients. One of the latter complained to her on one of her visits that she had been lying in the same place for several hours and had not had a change. She said that she wished God would remove her. She had met two patients in the Melbourne Hospital who had left the Austin Hospital because of their treatment, and one of them, a lad, said he would sooner die than return. Only last week a patient had told her that he had to leave because the Matron had taken a spite against him and had stopped his tobacco and eggs. She was not troubled with the same complaints about the Matron in the other institutions which she visited, such as the Melbourne, Women's, and Homœopathic hospitals.

Perhaps the most damaging statement was that made by a nurse: "Mrs. Harvey stated that she had been neglected by the nurses, and had been driven to buying medicines outside the institution for herself. Nurse Turner stated that she was unable to apply the remedial measures prescribed by the doctor every day as she had not sufficient time at her disposal. The remedies were applied every alternate day." In conclusion, I need only recall to your readers' minds the fact that the Matron, in giving evidence before the Charities Committee, stated that trained nurses were not necessary for incurables. I went over the Austin Hospital a short time since, and was astonished to find that the Matron knew nothing of some of the patients, though to me, a stranger, their cases were peculiarly interesting.

The Charities Commission report is out, and so far as it deals with nursing is sadly inadequate and disappointing. They recommend: (1) The employment of female nurses in hospitals wherever practicable; (2) the establishment of a board from which nurses should obtain a certificate of competency; (3) the provision of better accommodation for nurses, relief from menial work, and the raising of their status. The only hint they give of a practical nature is this: "In reference to female nurses, it is suggested that for the purposes of examination a competent authority will require to be called into existence. It should consist of a board of nine members, of whom three should be nominated by the whole body of the trained and certificated nurses of the colony." Of course, the only competent authority to judge of a nurse's merit is the Matron under whom she was trained. A woman who knows the names of all the bones of the skeleton, and can read a prescription, might pass an examination, and yet be an abominably bad nurse, lacking the skilful hands, the cheerful temper, the knowledge of minute detail that makes a sick man comfortable. We were just beginning to go ahead here nicely in the nursing line, and if any attention is paid to the above recommendations, it will be a terribly retrograde step. At the December meeting of the Melbourne Hospital, the Matron reported that the newly-established nursing school was succeeding wonderfully well: Dr. William Moore had just completed an interesting and instructive course of fifteen lectures on surgical nursing and anatomy, and an examination of the student nurses in these subjects was now being conducted by Dr. Moore and Dr. Molloy. Now, are not the proper people to examine and grant certificates to these nurses, those who not only know their theoretical knowledge, but have seen their practical work in the wards? Also take the following report from the December meeting of the Alfred Hospital: "The Matron (Miss M. D. Farquharson) presented the half-yearly report of the nurses' training school and staff, showing that seven pupils for preliminary, and two for final, examination had passed the examiners' test. During the late epidemic a great strain had been placed on the nurses who escaped the influenza, so much extra work being required from them. This was borne without a murmur, and no extra nurses from outside were engaged." It will, indeed, be hard if these schools are to be overridden by an outside authority, especially considering the elementary state of nursing here, and the style of some women who claim to be trained nurses.

The fourth session of the Australasian Association for the Advancement of Science was began in Hobart on January 7th, and has been pronounced, so far, to be a decided success. Indeed, the members of the Association, who have attended the whole of its meetings since its happy inauguration in New South Wales, are agreed that the present session is the most notable that has been held. Papers are being read fast and furiously, the most popular subject being the proposed Antarctic expedition. Medical subjects are rather left out in the cold.

The Morgue returns for the year show a shocking practice of infanticide in this city. Within the city coroner's jurisdiction alone 34 infants have been either accidentally or purposely killed during the year; in 17 cases verdicts of wilful murder were returned, and in not one solitary instance were the murderers brought to punishment.

Dr. Beaney's brother and sister have made a peculiar request for money from the legacies left by him to various hospitals. They state that they are destitute. In most cases the request has been passed over.

The *Argus*, our chief daily paper, distributed last year £11,000, being money received in answer to appeals in its pages.

At the annual meeting just held of the Medical Society of Victoria, Dr. Hinchcliff, in his retiring address, tried to rouse the Society to interfere in the present scandalous method of electing medical officers to hospitals by subscribers' votes; he also attacked the club system strongly.

### Wants and Workers.

*Acknowledgment.*—Miss Middleton and Nurse Gates thank those who have answered their appeal. They now have £24, and hope to shortly remove the nurse to her new home. A few more annual subscriptions would be welcome.

### The Queen's Nurses.

On February 24th, at Westminster Hospital, the Rev. the Master of St. Katherine's, read a paper before the Hospitals Association on the "Queen Victoria Jubilee Institute for Nurses: The Object and the Work." Mr. Bryant, President of the Royal College of Surgeons, was in the chair, and there was a large attendance of nurses wearing the uniforms of the different district associations. Mr. Peile first gave a short account of how the Institute was started by the women's jubilee offering to Her Majesty, and then went on to give a brief history of St. Katherine's, where the work has its centre and its home. Coming to the more interesting part of his paper, the work itself, Mr. Peile said he had noticed in nursing papers an expression of dislike to the fact that would-be probationers were requested to apply to the Secretary. He wished to state that this was merely done because it was more convenient for all correspondence to come to one office, and that letters directly concerning the nurses were at once handed over to the Inspector, who was herself a trained nurse of great experience. The standard of training for the nurses is one year in a good general hospital and six months in district nursing; also midwifery training is insisted on if the applicant is going to a country post. Those nurses trained by the Institute in district nursing have to promise to serve for two years.

There are now nine homes in which the Institute is able to train, and it has trained in England 32 women, in Scotland 51, and in Ireland 10. The title of "Queen's Nurse" can only be held by a woman working for a home affiliated with the Jubilee Institute, and who has fulfilled the conditions of training. She can then, on the recommendation of the Council, be enrolled as a Queen's nurse, upon which she is presented with a badge to be worn on the left arm. There is also a badge for Superintendents, and one for special service; the latter has been given by Her Majesty to the late Inspector, Miss Rosalind Paget.

Mr. Peile went on to state that one of the houses of St. Katherine had lately become vacant, and would shortly be opened as a home for the nurses, where they could spend a few days free of charge, in certainly not the dreariest part of London. The fact that the work of the Institute is absolutely unsectarian was strictly enforced, and was shown by the fact that in Dublin there are two training homes, one for Catholics and one for Protestants, but both under the superintendence of Miss Dunn. With regard to affiliation, Mr. Piele pointed out that the Institute did not interfere with the work of local committees, it simply, through the Inspector, saw that the proper standard of nursing was maintained, and it aided the affiliated home in many ways. The activity of the Scottish Branch, and the slow progress in Ireland were referred to, also the fact that the Rural Branch has now 32 nurses at work.

Dr. Steele rose at the conclusion of the paper and asked a very practical question as to the remuneration of the nurses. In reply the Master said that they began at £25 and all found, but as a rule they received £30 or £35.

Surgeon-Colonel Ince made a few rambling remarks, and then one of Miss Broadwood's nurses got up and stated she was paid £26, and gave some particulars as to the number of her family.

The Rev. J. D. K. Mahomet asked whether it was better for nurses to live in a central home or in lodgings, and one of the nurses present stated that it was much nicer not to have your own housekeeping to do.

Mr. Bryant then rose to ask where the district nurse got her instructions, and told a highly amusing tale of a nurse who went to a district, not fifty miles from London, and found herself the slave of two fussy old ladies, who sent her only to their own protégés. On seeing the nurse come out of a cottage to which the doctor had sent her, one of the old ladies said, "What were you doing in that cottage? I never sent you there. Those are miserably poor people; very bad people. You are to have nothing to do with them." Mr. Bryant told the story very well, and roused the laughter of the audience, and also succeeded in bringing Miss Paget to her feet, who said that the Queen's nurses were only allowed to attend cases which were under a doctor's charge, and that they were sent to the cases by the Superintendent. Miss Paget added that the nurse in the above story had her ladies in very bad order, and must have been wanting in tact. She usually found the old ladies very obedient to the nurses, though, of course, having given their money to the scheme, they liked to think they had a finger in the pie. Dr. Steele proposed a vote of thanks for the use of the room, and coupled with it the name of Mr. Quennell; and Dr. Gilbart Smith proposed a vote of thanks to the Chairman, and then the meeting broke up.

### Everybody's Opinion.

[*Correspondence on all subjects is invited, but we cannot in any way be responsible for the opinions expressed by our correspondents. No communications can be entertained if the name and address of the correspondent is not given, or unless one side of the paper only be written on.*]

### A NURSING RESERVE.

"An Irish Sister" writes: May I intrude on your valuable time by writing a few lines in answer to "Red Cape." She wishes to know if the idea of a Reserve Nursing Staff should be thought practicable. I should think, if I may venture my humble opinion, that it would be a most advisable scheme, and I am sure would meet with the wishes and desire of many of the nurses and Sisters of Great Britain. I myself applied a little time ago to the War Office to know if such a staff were in existence, but was as answer returned the forms to fill up to enter as probationer Netley Hospital (which, after six years' nursing, I did not feel inclined to do) as the only means of service in case of war. So I should think "Red Cape's" 'suggestion should be the very thing to start. Trusting that before long it will be a really working branch of nursing, and am sure many will be only too glad to join.

### Appointments.

Braintree Cottage Hospital.—Miss Laura King has been elected Matron of this hospital. She trained at the Essex and Colchester Hospital for four years, and for two years served the St. Albans Diocesan Nursing Institution. Her testimonials are excellent, and she deserves her promotion.

Hope Hospital.—Miss A. G. Sibley has been appointed Matron of this hospital. At present Miss Sibley is Matron of the Worthington Hospital, to which she was elected in 1888, after having held the post of night Sister at St. Mary Abbott's Infirmary. We hope Miss Worthington will be successful in the difficult post she has undertaken to fill.

Northampton Infirmary.—Miss Marian Neepe has been appointed to the important post of Superintendent of Nurses at this infirmary. Miss Neepe trained at the London Hospital and passed excellently at the examinations; she also holds the Pendlebury Certificate. For three years Miss Neepe acted as Sister at Charing Cross, and lately has been working at Wigan as Night Superintendent. We congratulate her on her steady rise in her profession.

Yeatman Hospital, Sherborne—Miss M. Jennings has been appointed Matron of the Yeatman Hospital, Sherborne, out of a large number of candidates. Miss Jennings was trained at the Bristol General Hospital, and has for the last two years and a half held the post of Sister of an important male surgical ward in the South Devon Hospital, Plymouth, in which position she has gained the esteem, and leaves with the sincere regret and good wishes of those with whom she has worked. The Yeatman Hospital is to be congratulated on securing her services.

### Nursing Uniforms.

### I.—HER MAJESTY'S NURSING SISTERS.

Amongst the most picturesque of all nursing uniforms are those worn by the Army and Navy Sisters, and which are pictured above. The Army Sister wears a grey biege gown, a white apron with bib, and scarlet cape, and a white cap tying under the chin. When Miss Wheldon, of Netley, received the Royal Red Cross from the Queen's own hands, Her Majesty, touching the grey frock, remarked, "I like this stuff very much, but you ought to wear washing material." "May it please your Majesty," answered Miss Wheldon promptly, "it washes excellently;" and, in truth, if nursing Sisters do wear woollen materials, they ought always to be washable. When on active service the Army Sisters wear a plain handkerchief cap, folded three-corner ways, one point hanging down behind, the other two pinned under the hair. In hot climates, also, white cotton frocks are allowed to supplant the grey biege. The outdoor uniform consists of a circular grey cloak, small grey bonnet, and grey veil; amongst soldiers the Sisters are generally known as "The Grey Sisters," owing to the colour of their uniform. In the above illustration the Army Sister is represented as wearing the Royal Red Cross, the Soudan Medal, and the Khedive's Bronze Star, particulars of which decorations with lists of holders were given in our pages for December 6th, 1890, February 1st, 1891, and May 9th, 1891.

The Naval Nursing Sisters wear a navy-blue gown and cape with scarlet facings, on the left arm also they wear a small scarlet cross on a white ground. The cap is white, and ties under the chin, but it differs from the military cap in having two goffered frills. The apron is white with a bib. Out of doors the Navy Sisters wear a blue Mother Hubbard cloak, faced with scarlet, which is very becoming, neat little navy-blue bonnets and veils.

We propose to discuss week by week a few of the most notable nursing uniforms, dwelling on their comparative advantages and disadvantages, and giving illustrations. We have not, however, the audacity to criticise the uniforms of "the services."

### Death in our Ranks.

On December 8th, 1891, Helena Alfreda Peckham, aged 33. Nurse H. A. Peckham trained at Guy's, and worked in connection with that hospital for nine years. She died of consumption.

The Dover Nurses' Institution has sustained a grievous loss in the death of Nurse Agnes, who joined it in April, 1878. Her skill and experience and loyal devotion to her duty made her a blessing to every patient whom she nursed, either rich or poor, and her unfailing common sense and unselfishness were the greatest help in every house of sickness. Her bright and kindly disposition endeared her to her fellow nurses and to all with whom she worked. She died at Brenchley on February 25th, of enteritis, after only five days' illness. She was conscious to the time of her death, and to the very end her thoughts were (as they had been through her life) for others, not herself.

### Betty's Angel.

It was quite a festival in the little top-floor room that belonged to Widow Beamish. Not that anything could make the floor and walls look bright—they were too begrimed with time-honoured dirt-stains ; but what could be done had been done by the tenant, in preparation for a joyous event, the return of her child Betty from hospital. Betty Beamish had waded through a bad spell of fever, poor little soul; indeed, there had been nights and days when nobody hoped Betty would ever leave the hospital walking on her own small feet. But the end of the gloomy wood was reached at last, and Betty was out in the open of returning convalescence. Then arrived the going-home day, and though it had seemed as if it would never come, mother and Betty found themselves, radiant and over-joyed, sitting close up to the fire, their feet planted comfortably on the fender, and the tea-table decked out lavishly, in honour of the occasion, with bread and butter and watercresses.

"It be rare and sweet, Betty, to have 'ee back once agin," said mother, as she leaned over to stroke lovingly the close-cropped, little yellow head—for Betty's hair had been shaved off after the fever. There were but two in the home, mother and Betty, and their hearts were knit together. The child was not exactly like other children ; some said she was a "natural," meaning that her wits were a bit scattered; others, again, called Betty an innocent. Perhaps it would be well if this world were made up of such innocents. "Now, tell me, deary," went on mother, watching with pride how the convalescent appreciated the prepared feast; "was they real good to 'ee at the 'orsepital?"

Betty laid down her bread and butter, and turned her dreamy, grey eyes upon her mother's face.

"Just real good, mother!" she spoke, with softly-smiling lips. "All of them was lovin' to me. And, mother, there was a nangel came most nights, with such a beauty face, and white wings flying out behind her shoulders. *She* was best of all."

"An angel! wasn't it a nuss? And, happen, the wings were the long white streamers of her cap?"

"No," declared Betty stoutly, "it was my nangel. An' the night afore I left, last night that is, she kissed me good-bye; an' I says, says I, 'Be you going home to heaven now, please?' Says she, 'I hope I be; an' if I go first, Betty, I'll look out for your little figure toiling up the steeps to the gates of the Golden City!'" The child ceased, and stared absently into the fire.

"Don't, Betty, don't look like that!" cried the affrighted mother. "You be to stay along o' me on earth. I can't do without 'ee. Now, look 'ee here. Grandmother, she was here last Sunday was a week, and she left a penny for 'ee, with her love."

Betty's fragile fingers toyed with the coin, but she continued to gaze into the fire, smiling dreamily and planing out, perhaps, how to lay out her wealth.

A large house in a once-upon-a-time fashionable street, the home of a hard-working parish priest. The good man had a house-full of daughters, girls who must and did work; one taught; another wrote; a third was a trained nurse—Betty Beamish's Angel, the Nurse Amy who had so lovingly tended the fever-stricken mite, nursing her into convalescence, and then, lying down, herself a victim to the same fever.

"Take me home to be ill!" she had prayed, and they did so.

The dreadful fever had raged itself out; the patient lay spent, and helpless. It was a question whether she had enough vitality left to allow hope of recovery. There was much coming and going in the agitated household. At the open street-door stood a quaint, small figure, all wide eyes and white pinafore; in its hand, clutched tightly, a bunch of violets just purchased. It was Betty who had discovered the priest of mother's church to be the father of her angel. Watching her opportunity, the child slipped into the house and up the stairs. With strange instinct—but then Betty was a natural—she pushed open the right door. In the shaded light she was unnoticed as she stole to the bed-side by all save one pair of heavy eyes that regarded her with a sudden dawning interest.

"I be come to nuss you," said Betty, calmly seating herself, and softly patting the patient's brow as she placed the dewy violets exactly beneath the wasted chin; "I've put on a clean pinny, but I haven't got a cap, does it matter?" she added anxiously.

A feeble quiver—it must have been laughter's ghost—stole round the white lips of Nurse Amy; then, as somebody tried to hustle the new-comer from the room, a disturbed look came into her face.

"Let the child stay," said somebody else who was wiser; and Betty stayed to nurse her angel.

Nurse Amy is in harness again; and Widow Beamish finds her little daughter growing up to be a famous help. Betty and her angel are fast friends, for they never forget that they nursed each other through just the same fever; at least, that's what Betty says about it.

### East London Nurses.

The Lord Mayor took the chair at the annual meeting of the East London District Nursing Society, and a large gathering of friends mustered at the Guildhall. There was Mrs. Stuart Wortley, the Rev. Harry Jones, the Matrons and nurses. The speeches were mostly clerical in tone, but relieved by the practical utterances of Mr. Tennant and Mr. Cursham Corner. The Lord Mayor not only spoke enthusiastically in favour of this society, but undertook to try and get it a grant of money. Truly the society is one of the best with which we are acquainted, but we could wish that its admirers amongst the clergy would not praise it at the expense of other charities; it is not Christian to say the least of it. None of the speakers seem to have heard of the proposed district nurses under the Local Government Board.

Mr. Percy Wigram writes on this subject: "I have no wish to belittle the admirable work done by the North London Nursing Association, but I must demur on the part of the East London Nursing Society to two statements made in the 'Nursing Mirror' for February 20th—viz. (1) That they were the first branch of the Metropolitan and National Nursing Association, and (2) that they are doing the largest work of the kind in London. The East London Nursing Society was formed in 1868. Its supporters took an active part in the foundation of the Metropolitan and National Nursing Association in 1876, and it became the eastern branch of that body. In 1881 it, like the North London Nursing Association, resumed a separate existence. Under these circumstances it may, I think, fairly claim to have been the first branch. As to the amount of work done, I have no report of the North London Nursing Association later than 1889 before me, but I may take that as a fair example, as I see elsewhere that the number of nurses has not increased. Now in 1889 the North London Nursing Association, with a staff of 1 Superintendent and 9 nurses, attended 1,438 cases, and paid 29,672 visits; whilst the East London Nursing Society with 4 Matrons and 25 nurses had 3,645 cases, and 82,535 visits. Our expenses, making allowance for rent of lodgings, which is in most cases supplied by the districts, and therefore does not appear in our accounts, were almost exactly double those of the North London Nursing Association, but the work done exceeds that proportion. And since 1889 we have slightly extended, and now have 28 nurses." The report, which was submitted at the meeting, was in every way satisfactory and very interesting.

## Figures and Tables

**Figure f1:**
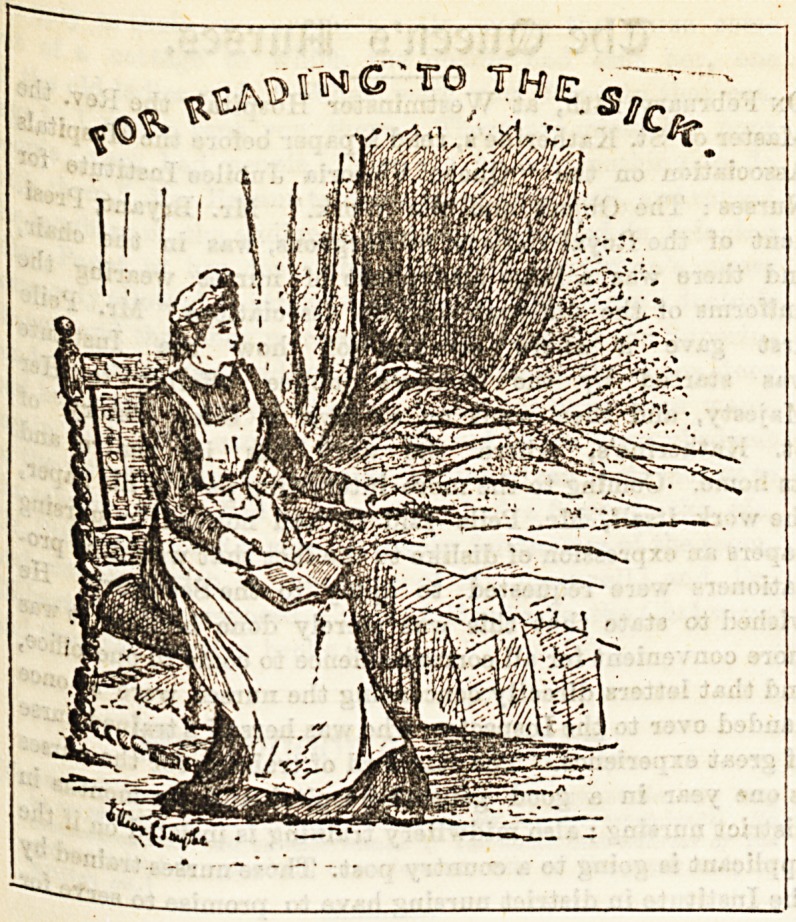


**Figure f2:**